# Monoclonal Antibodies in Prevention and Early Treatment of COVID-19 in Lung Transplant Recipients: A Systematic Review and Perspective on the Role of Monoclonal Antibodies in the Future

**DOI:** 10.3389/ti.2025.13800

**Published:** 2025-02-10

**Authors:** David A. Van Eijndhoven, Robin Vos, Saskia Bos

**Affiliations:** ^1^ Medical School, Catholic University Leuven, Leuven, Belgium; ^2^ Department of Respiratory Medicine, University Hospitals Leuven, Leuven, Belgium; ^3^ Department of CHROMETA, Laboratory of Respiratory Diseases and Thoracic Surgery (BREATHE), KU Leuven, Leuven, Belgium

**Keywords:** lung transplantation, COVID-19, Sars-CoV-2, monoclonal antibodies, tixagevimab/cilgavimab, sotrovimab, casirivimab/imdevimab, bamlanivimab

## Abstract

Coronavirus disease 2019 (COVID-19) has significantly impacted lung transplant recipients (LTR), who remain vulnerable to severe COVID-19 despite vaccination, prompting the use of monoclonal antibodies (mAbs) as a treatment option. This systematic review summarizes the clinical efficacy of mAbs against COVID-19 in adult LTR and provides a perspective on the role of mAbs for infectious diseases in the future. A systematic search of PubMed/MEDLINE, Embase and Cochrane was conducted for studies reporting clinical outcomes of adult LTR or solid organ transplant recipients (SOTR) including LTR with drug-specific outcomes. Twelve studies were included. Pre-exposure prophylaxis with mAbs reduced COVID-19 breakthrough infection in LTR. Early treatment of COVID-19 with mAbs correlated with a reduced incidence of severe COVID-19 outcomes, although statistical significance varied among studies. Overall, observational studies have demonstrated a potential benefit of mAbs in the treatment of COVID-19 in LTR, both in prophylaxis and early treatment, as well as the importance of early administration. Moreover, mAb therapy appeared safe and could be a viable option against other pathogens, a route that warrants further investigation.

**Systematic Review Registration:**

https://www.crd.york.ac.uk/prospero/display_record.php?RecordID=382133, identifier CRD42022382133.

## Graphical Abstract

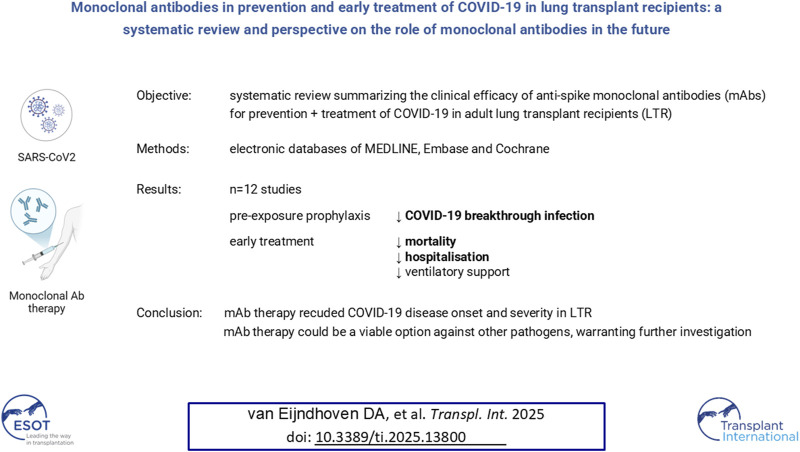

## Introduction

Since its emergence in 2019, severe acute respiratory syndrome coronavirus 2 (SARS- CoV-2) significantly affected the field of organ transplantation. Solid organ transplant recipients (SOTR) are more susceptible to severe coronavirus disease 19 (COVID-19) outcomes compared to the general population, resulting in increased hospital admissions and mortality [1–3]. This is mainly due to a higher occurrence of underlying comorbidities and the use of immunosuppressive therapies in SOTR [3, 4]. Lung transplant recipients (LTR) in particular are at increased risk of severe COVID-19 compared to other SOTR [5–7]. Although mortality and hospitalization rates have decreased, LTR are still at elevated risk of severe COVID-19–related morbidity and mortality [8, 9].

Vaccination is a key element in the prevention of severe COVID-19. However, LTR have a lower antibody response compared to the general population, even after receiving multiple vaccinations [8–10]. The number of COVID-19 breakthrough infections after vaccination have been significantly higher in LTR compared to other SOTR [8, 10, 11]. Meanwhile, other prophylactic and therapeutic agents have been repurposed and developed to prevent and treat COVID-19.

Monoclonal antibody (mAb) therapy has been a promising treatment option for COVID-19. Multiple randomized controlled trials have reported reduced COVID-19-related hospitalization or death after administration of mAbs [12–16]. However, these studies were primarily focused on immunocompetent patients in an outpatient setting. Nevertheless, multiple mAbs received emergency use authorization for COVID-19 treatment in high-risk patients, including LTR. Subsequently, retrospective cohort studies reported decreased COVID-19-related hospitalization and mortality rates in SOTR after treatment with mAbs. Since then, mAbs have commonly been used for therapeutic management in SOTR [17, 18] However, the emergence of new SARS-CoV-2 variants has diminished the neutralizing efficacy of mAbs used early in the pandemic [17, 19]. Nevertheless, LTR and similar high-risk patients with weak post-vaccination antibody responses may still benefit from mAb therapy [4, 8, 10].

While multiple retrospective cohort studies reported use of mAbs in SOTR [20–22], data specifically about mAbs against COVID-19 in LTR remain scarce, even though LTR are identified as a high-risk group [6–10]. This systemic review aimed to describe the existing evidence pertaining the impact of anti-spike mAbs used for prevention and treatment of COVID-19 on clinical outcomes of adult LTR in two modalities: pre-exposure prophylaxis (PrEP) and early treatment in LTR with asymptomatic to moderate COVID-19.

## Methods

This systematic review was performed according to the Preferred Reporting Items for Systematic Reviews and Meta-Analyses (PRISMA) 2020 guidelines [23]. A protocol for this review was registered on the PROSPERO International Prospective Register of systematic reviews (CRD42022382133).

### Search Strategy and Eligibility Criteria

A systemic search on the databases of PubMed/MEDLINE, Embase and Cochrane Controlled Trials Register (CENTRAL/CCTR) was performed on 8th February 2023. The used search terms are listed in the Supplementary Material. Clinically commonly used COVID-19-specific, anti-spike mAbs were included. The following mAbs were included: tixagevimab/cilgavimab, sotrovimab, casirivimab/imdevimab, bamlanivimab, bamlanivimab/etesevimab, regdanvimab, bebtelovimab, and sarilumab.

The articles were imported into Rayyan [24]. The abstracts and titles were independently screened by two reviewers (DV, SB) using predefined inclusion and exclusion criteria, followed by full-text review if potentially eligible for inclusion. Discrepancies were resolved by consensus.

Eligibility criteria were defined beforehand. The initial inclusion criteria were studies containing clinical outcomes on adult LTR after administration of mAbs, with drug-specific outcomes. Since only a limited number of studies reported LTR-specific data, we subsequently broadened the inclusion criteria to cohorts of SOTR that also included LTR [so only combined groups of SOTR, other organ transplant-specific outcomes (e.g., kidney transplant population) were not included]. Eligible studies included any randomized controlled trials, prospective and retrospective observational cohort studies, case series, and letters to the editor if they included clear data analysis. Conference abstracts, case reports, reviews, letters to the editor without separate data analysis, and non-English articles were excluded. No time restrictions were applied.

### Data Collection Process and Items

One reviewer (DV) performed data extraction using a standardized data extraction form that was inspected by a second reviewer (SB). From each included study we extracted study properties, patient characteristics, therapeutic regimen, and outcomes. Main outcomes were overall mortality and COVID-19-related mortality. Additional outcomes were defined as incidence of hospital admission, intensive care unit (ICU) admission, necessity of respiratory support (defined as high-flow nasal oxygen, non- invasive ventilation or mechanical ventilation), secondary complications (bacterial and fungal secondary infection, renal insufficiency, and venous thromboembolism), and long-term lung function data.

### Risk of Bias Assessment

One reviewer (SB) performed a risk of bias assessment using the revised Cochrane risk- of-bias tool for randomized trials [25] or the Newcastle-Ottowa Scale [26] for non- randomized trials (including case control and cohort studies).

### Data Synthesis

Random-effects meta-analyses would be performed if the extracted outcomes were clinically and statistically feasible for pooled analysis. However, due to significant heterogeneity across the included studies, data could not be pooled for meta-analyses. Outcomes are reported per mAb in the evidence profiles (Supplementary Material).

## Results

### Literature Search

**FIGURE 1:**
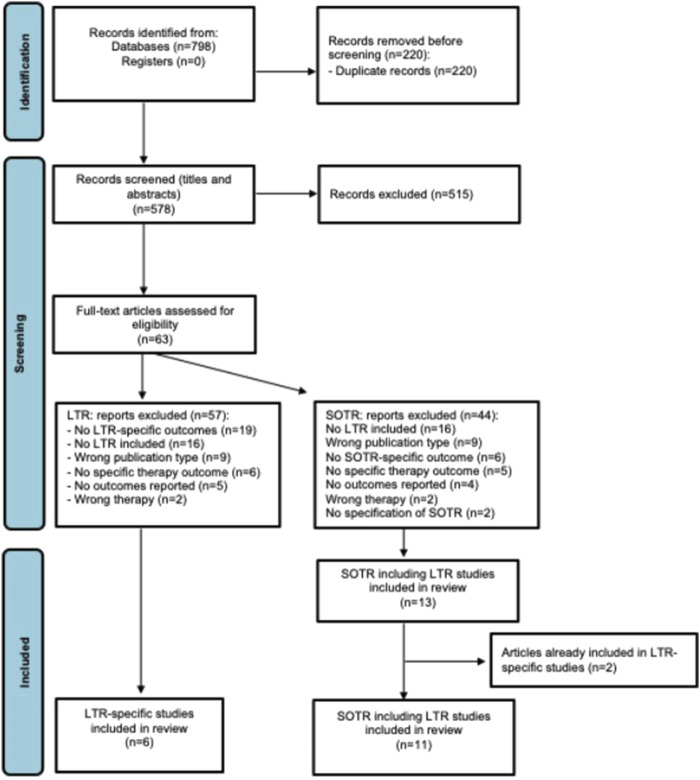
PRISMA flow diagram of included studies. LTR, lung transplant recipients; SOTR, solid organ transplant recipients.

The database searches yielded 798 articles. After removing 220 duplicates, 578 studies were screened by title and abstract. Sixty-three papers were assessed for full-text eligibility with 43 articles excluded. Reasons for exclusion are summarized in Figure 1. Subsequently, results for tocilizumab, a non-COVID-19-specific mAb, were excluded as well as to include only data on anti-spike mAbs. In total, three studies with LTR-specific outcomes [27–29] were included and nine articles with SOTR-specific outcomes that included LTR [30–38].

mAbs were given as PrEP in four studies [27, 28, 30, 31] and as early treatment in LTR with asymptomatic to moderate COVID-19 in nine studies [29, 31–38]. No data on bamlanivimab/etesevimab, regdanvimab and sarilumab were found in our specific population. In terms of risk of bias analyses, most outcomes had an intermediate risk of bias, meaning that there were some concerns in at least one domain in the risk-of-bias judgement for a specific outcome. Additional information can be found in the evidence profiles in the Supplementary Material.

### Pre-Exposure Prophylaxis

Studies that included LTR who were not infected with COVID-19 at the time of mAb administration.

#### Tixagevimab and Cilgavimab

Four studies were included in which tixagevimab/cilgavimab was administered as PrEP against COVID-19 in an outpatient setting [27, 28, 30, 31]. Vaccination coverage among the studies was high (94%–100%) [27, 28, 30]. Most common SARS-CoV-2 variants were Omicron B.1.1.529 [27], BA.4, BA.5 [30, 31] and BA 2 [27, 30, 31].

##### LTR-Specific Outcomes

Tixagevimab/cilgavimab was used in one matched cohort study (n = 444, including 77 LTR who were treated with PrEP and compared with 70 matched LTR) [27], and a retrospective cohort study (n = 1,112, which included 36 LTR) [28].

Both studies reported a rate of breakthrough COVID-19 infection of 8% for LTR treated with tixagevimab/cilgavimab [27, 28], which was significantly lower than that for the control group (8% vs. 23%, p = 0.010) [27]. In the matched cohort study, a higher (300/300 mg) dose was associated with a lower rate of breakthrough infection compared to low-dose PrEP (150/150 mg) (log-rank p = 0.025). A stratified analysis, considering the number of vaccines, indicated a reduced rate of breakthrough infections after treatment with tixagevimab/cilgavimab compared to the control group. This reduction was observed in SOTR with 0–3 vaccines (log-rank p = 0.006) and among those who received 4−5 vaccines (log-rank p = 0.008) [27]. Overall mortality for LTR was 0% in both studies [27, 28] with one LTR (1%) hospitalized in the study of Jurdi et al. [27]. The other study reported no need of respiratory support [28].

##### Outcomes From SOTR Studies

Two prospective studies evaluated the use of tixagevimab/cilgavimab in SOTR, consisting of one nationwide study (n = 392, including 54 LTR) [30] and one single-center study (n = 350, with PrEP administered to 205 SOTR) [31].

Breakthrough COVID-19 infections were low (8%–9%) [30, 31]. The nationwide study reported a higher infection rate for SOTR treated with a single dose of 150/150 mg of tixagevimab/cilgavimab (28%) compared to 300/300 mg (8%) or a double dose of 150/150 mg (0%) [30]. Incidences of mortality (0%–1%) and hospitalization (0.5%–1%) among SOTR were very low [30, 31], and no patients were admitted to the ICU or required respiratory support according to Alejo et al. [30].

### Early Treatment of COVID-19

Studies that reported SARS-CoV-2 positive LTR with asymptomatic to moderate disease according to the WHO scale receiving mAbs [39]. mAbs in early treatment consisted out of sotrovimab, casirivimab/imdevimab, bamlanivimab, and bebtelovimab.

#### Sotrovimab

Six studies used sotrovimab as early outpatient treatment after SARS-CoV-2 infection [29, 32–36]. During the study period, the predominant SARS-CoV-2 strain was Omicron BA.1 [29, 32, 35, 36], along with Omicron B.1.1 [32, 34–36] and Omicron BA.2 [29, 32, 33]. Vaccination coverage was moderate (53%–96% of SOTR received ≥3 SARS-CoV-2 vaccines) [32–35].

##### LTR-Specific Outcomes

One prospective cohort study reported 114 SARS-CoV-2-positive immunocompromised patients, including 16 LTR. Sotrovimab was initially only given to hospitalized patients. Due to high hospitalization rates, sotrovimab was subsequently implemented as an outpatient treatment for 14 LTR. Before outpatient treatment, 69% of LTR were hospitalized, 36% required at least 15 L/min or high-flow nasal oxygen therapy and one LTR (6%) died due to COVID-19. Administration in outpatient setting resulted in a significant reduction of hospital admissions [7% (11/16) versus 69% (1/14), p < 0.001]. Additionally, no LTR died after the implementation of outpatient therapy [29].

##### Outcomes From SOTR Studies

Five studies were included. In a prospective single-center cohort study by Solera et al. (n = 300), 106 SOTR, including 34 LTR, received sotrovimab and were compared to 187 SOTR, including 26 LTR [32]. A nationwide population-based study (n = 2,933) reported 800 SOTR (with 49 LTR and 2 heart-lung transplants), with 88% of SOTR receiving sotrovimab in outpatient setting and 12% during hospitalization [33]. Additionally, there were three retrospective cohort studies by Yetmar et al. (n = 361, with 260 SOTR, including 17 LTR) [34], Hedvat et al. (n = 154, of whom 51 SOTR, including 4 LTR) [35] and Cochran et al. (n = 88, including 18 LTR) [36].

Hedvat et al. and Solera et al. reported a lower incidence of overall mortality in SOTR with sotrovimab compared to their controls [0/51 (0%) versus 3/75 (4%) and 0/106 (0%) versus 12/187 (6%), respectively] [32, 35]. The remaining studies also reported a low mortality incidence (0%–1%) after sotrovimab [33, 34, 36]. Mortality due to COVID-19 was lower in the intervention cohort than in the control group of Hedvat et al. (0% versus 4%) [35]. Delayed admission of sotrovimab (≤3 days versus >3 days after positive test) was significantly associated with increased mortality in the study of Rasmussen et al. [multivariate hazard ratio 4.88 (95% CI: 0.59–1.83)] [33].

Sotrovimab significantly reduced COVID-19-related hospitalization and mortality rates in SOTR [10% (5/51) versus 31% (23/75) in controls, p = 0.007)] with a similar trend in overall mortality and hospitalization [12% (6/51) versus 33% (25/75), p = 0.009]. After adjusting for organ transplant type, sotrovimab was associated with a lower risk of 30-day hospitalization or death [adjusted relative risk 0.15 (95% CI: 0.05–0.47)] [35]. Solera et al. also noted a lower incidence of hospital admission after sotrovimab compared to the control cohort, although this was not statistically significant [16% versus 28%, relative risk 0.58 (95% CI: 0.59–1.83)]. However, the median hospitalization duration was significantly shorter in the intervention group (4 versus 7 days) (p = 0.002) [32]. Hospital admission in the remaining studies varied between 3% and 23% [33, 34, 36].

In the studies with control groups, no SOTR treated with sotrovimab required mechanical ventilation versus 5%–8% of control SOTR [32, 35]. Similarly, Cochran et al. found no need for respiratory support in 88 SOTR after sotrovimab [36]. Secondary infections occurred in 8% of the sotrovimab group and 15% in the control group [32]. Acute kidney injury was less frequent in the intervention cohorts, but differences were not statistically significant [10% versus 28% (p = 0.17) and 13% versus 21% (p = 0.12)] [32, 35].

#### Casirivimab and Imdevimab

Two retrospective single-center cohort studies included casirivimab/imdevimab as early treatment against COVID-19 in an outpatient setting. Both studies described solely SOTR- specific outcomes. COVID variant B.1.1.7 was dominant, however, no systematic testing and prevalence were reported. The studies were performed before SARS-CoV-2 vaccination implementation [37, 38].

##### Outcomes From SOTR Studies

Yetmar et al. reported the use of casirivimab/imdevimab in 18 SOTR (n = 73, including 2 LTR) [37], while Sarrell et al. compared 22 SOTR treated with casirivimab-imdevimab to 72 SOTR who did not receive mAbs (n = 165, including 13 LTR) [38].

No deaths occurred in the SOTR after casirivimab-imdevimab administration [37, 38] in contrast to 3% (2/72) in the comparator cohort of Sarell et al., with 1% (1/72) attributed to COVID-19 [38]. Hospital admission for SOTR treated with casirivimab-imdevimab ranged from 0% to 6% [37, 38], which was lower compared to the control group [15% (11/72) of SOTR hospitalized for COVID-19-directed therapy] [38].

None of the treated SOTR were admitted to ICU, compared to 1% (1/72) in the control cohort [38]. In both studies, no SOTR required respiratory support [37, 38]. Fewer patients required renal replacement therapy in the intervention group than in the control group (0% versus 9% of the hospitalized patients) [38].

#### Bamlanivimab

In the aforementioned studies of Yetmar et al. and Sarell et al., bamlanivimab was also used as early treatment against COVID-19 in the outpatient setting [37, 38]. No LTR-specific data were available.

##### Outcomes From SOTR Studies

Fifty-two SOTR were treated with bamlanivimab in the study of Yetmar et al. (n = 73) [37]. In the other retrospective cohort study (n = 165), 71 SOTR received bamlanivimab and were compared to 72 control SOTR [38].

Among the in total 126 SOTR treated with bamlanivimab, mortality rate was 0% versus 3% (2/72) in the control cohort of Sarrell et al., of which 1% (1/72) attributed to COVID-19 [37, 38]. The need for hospitalization for COVID-19-directed therapy was higher in the control group (15%) compared to SOTR treated with bamlanivimab (11%–13%), but this difference was not significant after age adjustment in the study of Sarell et al. [(95% CI: 0.18–1.32), p = 0.161] [37, 38]. Average length of hospital stay ranged from four to 7 days for bamlanivimab-treated SOTR [37, 38] versus 7 days in the control cohort [38]. Delayed administration of mAbs after COVID-19 symptom onset was associated with a higher incidence of hospitalization (p = 0.03) [37].

ICU admission occurred in 0%–3% in the bamlanivimab group versus 1% in controls, and 1% of the treated SOTR needed mechanical ventilation compared to 0% in controls [37, 38]. While Yetmar et al. reported no SOTR requiring respiratory support [37]. Among hospitalized SOTR, 75% of bamlanivimab-treated SOTR developed acute kidney injury, compared to 36% in the control group. However, no-one in the intervention group required renal replacement therapy, whereas 1% in the control cohort [38].

#### Bebtelovimab

No LTR-specific data were available. Two SOTR studies were included where bebtelovimab was used as early treatment in an outpatient setting [31, 34]. Omicron BA.2 [31, 34] predominated, accompanied by Omicron BA.5 [31] and B.1.1.527 [34]. Of the SOTR, 73% were fully vaccinated while 14% were unvaccinated according to Yetmar et al. [34].

##### Outcomes From SOTR Studies

Bebtolivimab was administered to 145 SOTR in one prospective single-center study (n = 300, including 18 LTR) [31] and to 92 SOTR (with 4 LTR) in a multicenter retrospective study (n = 361) [34].

The studies of Yetmar et al. and Cochran et a. showed a low overall mortality (0.7% and 2.0%, respectively) and hospitalization rate in bebtelovimab-treated SOTR (3% and 12%, respectively) [31, 34]. In the retrospective study, bebtelovimab treatment was not significantly associated with hospitalization (p > 0.99), whereas inadequate vaccination status was (p = 0.007) [34]. Cochran et al. reported no ICU admissions [31], and Yetmar et al. noted one case (0.7%) of mechanical ventilation during hospitalization [34].

## Discussion

This systematic review aimed to assess the efficacy of mAbs against COVID-19 in LTR. Despite the higher risk of severe COVID-19 in this population [6–10], specific studies pertaining the use of mAbs for LTR remain scarce. A summary of main findings is provided in Table 1.

**TABLE 1:** Main findings.

**Type of treatment**	**Type of studies**	**Main outcomes**
**Prophylaxis**		
- Tixagevimab/cilgavimab	LTR (n=2) SOTR with LTR (n=2)	- Low rate of breakthrough infections [18, 27, 30, 31]
		- Reduced breakthrough infections versus controls [27]
		- Reduced breakthrough infections with high- or double-dose PrEP versus low-dose PrEP [27, 30]
**Early treatment**		
- Sotrovimab	LTR (n=1) SOTR with LTR (n=5)	- Low mortality rate [29, 32–36] - Lower incidence of death versus controls [32, 35] - Early mAb administration was associated with reduced mortality [33] - Reduction in hospitalization rate [29, 32, 35] - Low need for respiratory support [32, 35, 36]
- Casirivimab/imdevimab	SOTR with LTR (n=2)	- Low mortality rate [37, 38] - Lower incidence of death versus controls [38] - Low hospitalization rate [37, 38] - Reduced incidence of hospitalization versus controls [38] - Low need for respiratory support [37, 38]
- Bamlanivimab	SOTR with LTR (n=2)	- Low mortality rate [37, 38] - Lower incidence of death versus controls [38] - No difference in incidence of hospitalization for COVID-19-directed therapy [37, 38] - Early mAb administration was associated with reduced incidence of hospitalization [37]
- Bebtelovimab	SOTR with LTR (n=2)	- Low mortality rate [31, 34] - Low hospitalization rate [31, 34] - mAb administration did not affect hospitalization

LTR, lung transplant recipients; mAb, monoclonal antibody; PrEP, pre-exposure prophylaxis; SOTR, solid organ transplant recipients. Respiratory support defined as high-flow nasal canula, non-invasive ventilation or mechanical ventilation.

Pre-exposure prophylaxis against COVID-19 was reported in four studies [27, 28, 30, 31] in which the use of tixagevimab/cilgavimab showed a reduction of COVID-19 breakthrough infection in LTR [27]. Other COVID-19-associated outcomes (e.g., ICU admission, mortality) were very low, with a not significantly lower incidence in the PrEP-treated cohorts [27, 28, 30, 31]. These findings align with recent studies showing lower morbidity and mortality in SOTR during the Omicron period with high vaccination rates [40–42]. Other reviews also reported reduced COVID-19 incidence and reduced COVID-19 complications (hospitalization, severe COVID-19 and mortality) in SOTR [9] and immunocompromised patients following the use of tixagevimab/cilgavimab [43]. Importantly, low-dose tixagevimab-cilgavimab was associated with a higher incidence of breakthrough infections [27, 30], supporting high-dose PrEP [44].

Early treatment of LTR with COVID-19 included sotrovimab, bebtelovimab, casirivimab- imdevimab, and bamlanivimab. Only one study reported LTR-specific outcomes in which sotrovimab was used for hospitalized and outpatient therapy with a significant reduction in hospitalization in case of outpatient therapy [29], emphasizing the importance of early treatment.

The remaining studies also suggested a positive trend in early mAbs treatment for SOTR, generally showing lower incidences of severe COVID-19 outcomes compared to SOTR not treated with mAbs. However, among the studies, these findings were inconsistent and not always statistically significant. Likewise, a recent meta-analysis reported a reduced likelihood in overall hospital admission and mortality after sotrovimab in SOTR with mild to moderate COVID-19 [45]. Similar benefits were observed in other retrospective studies, with decreased risks of severe respiratory illness [46] and hospitalization [47]. Importantly, two studies in our review showed that early administration of mAbs was associated with reduced hospitalization [37] and mortality [33], while another study showed shorter hospital stays [32], again highlighting the beneficial effect of prompt treatment. This was also shown in another recent study that showed that administration of mAbs as early treatment was associated with a lower risk of hospitalization or death in lung transplant recipients [48].

Initial RCTs deemed mAbs to be safe with minimal risk of serious and mild adverse events [12–16]. Multiple studies in our review concurred with these findings, reporting no to very low incidences of moderate to severe adverse events [27, 30, 31, 35, 36, 38]. Despite increased cardiovascular risk in SOTR, cardiovascular events after mAbs were rare (0%–2%) [27, 30, 31]. Importantly, allograft rejection was also rare with few to no episodes of rejection reported [30, 35, 36, 38]. Concluding that mAbs are well tolerated without evidence of increased risk of severe adverse events or allograft dysfunction.

### Perspective on the Role of Monoclonal Antibodies in LTR in the Future

The COVID-19 pandemic has shown that swift development of mAb therapy was possible for emerging viruses, resulting in efficacious treatments with acceptable safety profiles. This fosters exploration of near-future development of mAbs against other virulent pathogens for LTR and other immunocompromised patients.

mAbs are laboratory-made proteins, produced from a cell lineage created by cloning a unique white blood cell, that act like antibodies and attack specific epitopes on antigens.

Modern medicine is further revolutionizing towards personalized “tailored” therapy, adapted to individualized specific disease characteristics. In theory, mAb can be produced to bind to virtually any suitable target and current mAb production can produce human/humanized mAbs, minimizing the risks originally associated with their predecessors. Another advantage is that mAb therapy, in comparison with vaccines, relies less on the patient’s immune response, which is crucial in patients receiving immunosuppressive treatment. Their mechanisms of action include direct cell toxicity, immune-mediated cell toxicity, vascular disruption, and modulation of the immune system. [49] Nevertheless, despite the advances made during the COVID-19 pandemic, current routine use of mAbs in infectious diseases remains limited, and these products are largely unavailable for the broader transplant community. The latter is crucial, since infections are very common among SOTR, especially LTR, and are difficult to prevent despite precautionary measures and sometimes with only limited treatment options available or with important risks of adverse events associated with systemic administration of antivirals (e.g., hemolytic anemia with ribavirin, skin reactions with oseltamivir, etc.). The COVID-19 pandemic has demonstrated that the field for mAb development is much wider and may be applicable to other viral infections for which there are currently no effective treatments, such as MERS, norovirus, Ebola virus, hantavirus, dengue virus, Zika virus, etc., or for which current therapies for prevention and treatment are suboptimal, such as cytomegalovirus and others. On a broader scope, lessons learned from the use of mAbs during the COVID-19 pandemic may therefore hopefully accelerate the development of novel, much-needed antibody drugs as therapeutic agents for transplant recipients, which should ideally be evaluated in well-designed randomized trials [50].

Our study encountered several limitations. First, scarcity of studies reporting outcomes specific to LTR, reflecting limited available data in this very specific patient population and underscoring the need for further research in this population. Moreover, all included studies were observational, with the majority being retrospective. Subgroup analyses in studies which included SOTR were not always present, necessitating caution when extrapolating these findings to LTR. Furthermore, follow-up periods were short (1–3 months), which could limit the incidence of long-term outcomes. Additionally, one outcome (long-term lung function data) was not reported in the included studies, although this might be of specific interest for the lung transplant population. Specific criteria (e.g., mAb administration, ICU admission) differed among nations and studies which could lead to distorted results. The heterogeneity of included studies, encompassing the stages of the COVID-19 pandemic with the emergence of different variants alongside the development of additional therapies and vaccinations, further complicated the independent assessment of efficacy of mAbs. Finally, the mAbs included in this study are currently not used due to limited efficacy against circulating variants [51–53]. Since March 2024, an emergency use authorization has been issued for pemivibart as PrEP in moderate to severely immunocompromised patients, including LTR [54, 55]. Further evaluation of the efficacy and safety of this biological in LTR has yet to be evaluated.

## Conclusion

mAb therapy was shown to be safe and beneficial in LTR for PrEP and early treatment of COVID-19 disease. While these mAb may currently not be effective anymore due to evolving SARS-CoV-2 variants, it demonstrates the utility of mAb therapies. This type of prophylaxis and treatment may also be very valuable for other pathogens, especially for immunocompromised populations at increased risk of infections and related complications and mortality, demonstrating the need for further research and development.

## Data Availability

The original contributions presented in the study are included in the article/Supplementary Material, further inquiries can be directed to the corresponding author.

## Abbreviations

COVID-19, coronavirus disease 2019; ICU, intensive care unit; LTR, lung transplant recipients; mAb, monoclonal antibody; PrEP, pre-exposure prophylaxis; SARS-CoV-2, severe acute respiratory syndrome coronavirus 2; SOTR, solid organ transplant recipients.
