# Supplementary material for: Monoclonal Antibodies in Prevention and Early Treatment of COVID-19 in Lung Transplant Recipients: A Systematic Review and Perspective on the Role of Monoclonal Antibodies in the Future
Source: Transpl Int. 2025 Feb 10;38:13800. doi: 10.3389/ti.2025.13800 (PMC11849561; doi:10.3389/ti.2025.13800)
Supplement: Supplementary file 1 [file DataSheet1.pdf]

Monoclonal antibodies in prevention and early treatment of COVID-19 in lung transplant recipients: a systematic review and perspective on the role of monoclonal antibodies in the future

Online supplement

Search strategy……… p.2
Summary of findings tables…………………………………………………………………………………………………….. p.3

1. Search strategy

Search terms used on 8th February 2023:

PubMed/MEDLINE

(("Lung Transplantation"[Mesh] OR lungtransplant*[tiab] OR ((lung OR pulmon*)AND transplant*[tiab]) OR ((lung OR pulmon*)AND allotransplant* [tiab]) OR (lung tissue AND transplant*[tiab])) OR("Organ Transplantation"[Mesh] OR organ transplant*[tiab] OR organ graft*[tiab] OR (solid AND organ AND transplant* [tiab]) OR SOT[tiab] OR organ allotransplant*[tiab])
AND
(Bamlanivimab[tiab] OR (Bamlanivimab AND etesevimab[tiab]) OR Regdanvimab[tiab] OR Regkirona[tiab] OR Ronapreve[tiab] OR (Casirivimab AND imdevimab[tiab]) OR Sotrovimab[tiab] OR Xevudy[tiab] OR Bebtelovimab[tiab] OR Evusheld[tiab] OR (Tixagevimab AND cilgavimab[tiab]) OR Tocilizumab[tiab] OR Sarilumab[tiab] OR Kevzara[tiab])

Embase

(('lung transplantation'/exp OR ‘lung transplant*’:ti,ab,kw OR ‘pulmonary transplant*’:ti,ab,kw OR ‘lung allotransplant*’:ti,ab,kw OR ’pulmonary allotransplant*’:ti,ab,kw OR ‘pulmonary allograft*’:ti,ab,kw) OR ('organ transplantation'/exp OR ‘organ transplant*’:ti,ab,kw OR ‘organ graft*’:ti,ab,kw OR ‘solid organ transplant*’:ti,ab,kw OR ‘SOT’:ti,ab,kw OR ‘organ allotransplant*’:ti,ab,kw))
AND
(‘Bamlanivimab’:ti,ab,kw OR ‘(Bamlanivimab AND etesevimab)’:ti,ab,kw OR ‘Regdanvimab’:ti,ab,kw OR ‘Regkirona’:ti,ab,kw OR ‘Ronapreve’:ti,ab,kw OR ‘(Casirivimab AND imdevimab)’:ti,ab,kw OR ‘Sotrovimab’:ti,ab,kw OR ‘Xevudy’:ti,ab,kw OR ‘Bebtelovimab’:ti,ab,kw OR ‘Evusheld’:ti,ab,kw OR ‘(Tixagevimab AND cilgavimab)’:ti,ab,kw OR ‘Tocilizumab’:ti,ab,kw OR ‘Sarilumab’:ti,ab,kw OR ‘Kevzara’:ti,ab,kw)

Cochrane Controlled Trials Register (CENTRAL/CCTR)

(([mh "Lung Transplantation"] OR (lungtransplant* OR ((lung OR pulmon*) AND transplant*) OR ((lung OR pulmon*) allotransplant*) OR (lung tissue AND transplant*) OR ((lung OR pulmon*) allograft)):ti,ab,kw) OR ([mh "Organ Transplantation"] OR (organ transplant* OR organ graft* OR (solid AND organ AND transplant*) OR SOT OR organ allotransplant*):ti,ab,kw))
AND
((“Bamlanivimab” OR “(Bamlanivimab AND etesevimab)” OR “Regdanvimab” OR “Regkirona” OR “Ronapreve” OR “(Casirivimab AND imdevimab)” OR “Sotrovimab” OR “Xevudy” OR “Bebtelovimab” OR “Evusheld” OR “(Tixagevimab AND cilgavimab)” OR “Tocilizumab” OR “Sarilumab” OR “Kevzara”):ti,ab,kw)

Search results for tocilizumab (a non-COVID-specific monoclonal antibody) were not included in the final manuscript.

2. Summary of findings tables

Tixagevimab-cilgavimab

Lung transplant-specific outcomes

Bibliography:

a) Jurdi AA, Morena L, Cote M, et al. Tixagevimab/Cilgavimab pre-exposure prophylaxis is associated with lower breakthrough infection risk in vaccinated solid organ transplant recipients during the Omicron Wave. 2022;22(12):3130-36.

b) Nguyen Y, Flahault A, Chavarot N, et al. Pre-exposure prophylaxis with tixagevimab and cilgavimab (Evusheld) for covid-19 among 1112 severely immunocompromised patients. Clinical Microbiology and Infection. 2022;28(12).

Quality assessment			N° of patients		Effect	
N° of studies	Study design	Risk of bias	Intervention	No intervention	Relative	Absolute
**Overall mortality**						
2[a,b]	Observational	Intermediate	113	70*	Mortality was 0% in both groups in the matched control study (0/77 vs. 0/70) and also 0% (0/36) in the other study.	
**COVID-19-related mortality**						
2[a,b]	Observational	Intermediate	113	70*	COVID-19-related mortality was 0% in both groups in the matched control study (0/77 vs. 0/70) and also 0% (0/36) in the other study.	
**Breakthrough COVID-19 infection**						
2[a,b]	Observational	Low	113	70*	Breakthrough COVID-19 infection was 8% (3/36) in the study by Nguyen et al. and significantly lower in the patients who received tixagevimab-cilgavimab in the matched control study (6/77 or 8% vs. 16/70 or 23%, p=0.010). Lower dose tixagevimab-cilgavimab was associated with higher breakthrough infection for SOTR (150/150 mg vs 300/300 mg) (p=0.025).	
**Hospital admission**						
1[a]	Observational	Intermediate	77	70*	1/77 (1%) treated with 150/150 mg single dose vs. 1/70 (1%) control LTR.	
**ICU admission**						
-	-	-	-		-	
**Additional respiratory support (HFNC, NIV, MV)**						
1[b]	Observational	Intermediate	36		0% (0/36) received non-invasive ventilation.	
**Incidence venous thromboembolism**						
-	-	-	-		-	
**Incidence secondary infections**						
-	-	-	-		-	

Incidence renal impairment				
-	-	-	-	-
**Long-term lung function data**				
-	-	-	-	-

a) Retrospective multicenter matched cohort study including 222 SOTR (77 LTR) who received tixagevimab-cilgavimab as pre-exposure prophylaxis compared to 222 matched controls (with 70 LTR). COVID-19 variant: Omicron B.1.1.529, BA.2 and BA.2.12.1.
b) Retrospective, multicenter study of 1112 immunocompromised patients, including 36 LTR, received tixagevimab-cilgavimab as pre-exposure prophylaxis. COVID-19 variant: Omicron BA.1 and BA.2.

[*] Study of Jurdy et al. matched cohort with 222 control SOTR including 70 LTR.

COVID-19: coronavirus 2019 disease, HFNC: high-flow nasale canula, MV: mechanical ventilation, NIV: non-invasive ventilation.

Solid organ transplant outcomes from studies that included lung transplant recipients

Bibliography:

a) Alejo JL, Kim JD, Chiang TP, et al. Patient-reported outcomes after Tixagevimab and Cilgavimab Pre-exposure prophylaxis among solid organ transplant recipients: Safety, effectiveness, and perceptions of risk. Clinical Transplantation. 2023;37(4).
b) Cochran W, Salto-Alejandre S, Barker L, et al. Covid-19 outcomes in solid organ transplant recipients who received Tixagevimab-cilgavimab prophylaxis and/or Bebtelovimab treatment in a nurse-driven monoclonal antibody program during the Omicron Surge. Transplantation. 2022;107(2).

Quality assessment			N° of patients		Effect	
**N° of studies**	**Study design**	**Risk of bias**	**Intervention**	**No intervention**	**Relative**	**Absolute**
**Overall mortality**						
1[a,b]	Observational	Intermediate	597		a) 0/392 (0%).<br>b) 2/205 (1%) (7% (1/14) in SOTR treated with 150/150 mg, 0.6% (1/156) in SOTR with 300/300 mg and none (0/35) in 150/150 mg double dose).	
**COVID-19-related mortality**						
1[a,b]	Observational	Intermediate	597		0% (0/392) and 2/205 (1%).	
**Breakthrough COVID-19 infection**						
1[a,b]	Observational	Low	597		a) 9% (36/392).<br>b) 16/205 (8%) (breakthrough COVID-19 infection was 29% (4/14) in SOTR treated with single dose 150/150 mg, 8% (12/156) in SOTR with 300/300 mg and none (0/35) in the cohort of double 150/150 mg dose).	
**Hospital admission**						
1[a,b]	Observational	Intermediate	597		a) 2/392 (0.5%).<br>b) 3/205 (1%) (tixagevimab-cilgavimab 150/150mg: 1 (7%), double 150/150mg: 0 (0%), single 300/300 mg: 2 (1%)).	
**ICU admission**						
1[a]	Observational	Intermediate	392		0/392 (0%).	

**Additional respiratory support (HFNC, NIV, MV)**				
1[a]	Observational	Intermediate	392	0/392 (0%) (NIV).
**Incidence venous thromboembolism**				
-	-	-	-	-
**Incidence secondary infections**				
-	-	-	-	-
**Incidence renal impairment**				
-	-	-	-	-
**Long-term lung function data**				
-	-	-	-	-

a) Nationwide prospective cohort study of 392 SOTR, including 54 LTR. COVID-19 variant: Omicron BA.2 and BA.4/5.

b) Retrospective single-center study of 350 SOTR, of whom 205 SOTR were treated with tixagevimab-cilgavimab. COVID-19 variant: Omicron BA.2 and BA.5.

COVID-19: coronavirus 2019 disease, HFNC: high-flow nasale canula, MV: mechanical ventilation, NIV: non-invasive ventilation.

Sotrovimab

Lung transplant-specific outcomes

Bibliography:

a) Malahe SR, Hoek RA, Dalm VA, Broers AE, et al. Clinical characteristics and outcomes of immunocompromised patients with coronavirus disease 2019 caused by the Omicron variant: A prospective, observational study. Clinical Infectious Diseases. 2022;76(3).

Quality assessment			N° of patients		Effect	
**N° of studies**	**Study design**	**Risk of bias**	**Intervention**	**No intervention**	**Relative**	**Absolute**
**Overall mortality**						
1[a]	Observational	Intermediate	14	16	Before sotrovimab outpatient treatment, 1/16[*] (6%) LTR died versus 0/14 (0%) after implementation of sotrovimab outpatient treatment.	
**COVID-19-related mortality**						
1[a]	Observational	Intermediate	14	16	Before sotrovimab outpatient treatment, 1/16[*] (6%) LTR died versus 0/14 (0%) after implementation of sotrovimab outpatient treatment.	
**Hospital admission**						
1[a]	Observational	Intermediate	14	16	Before sotrovimab administration in outpatient therapy, 69% of LTR (11/16) required hospital admission. 14 LTR received outpatient sotrovimab with 1 LTR requiring hospital admission (7%) (p= <0.001).	
**ICU admission**						
-	-	-	-	-	-	
**Additional respiratory support (HFNC, NIV, MV)**						
1[a]	Observational	Intermediate	NA	16	Before sotrovimab outpatient therapy, 4/11 (36%) who were hospitalized (25% of all 16 LTR[*]) required at least 15 L/min or high-flow nasal oxygen therapy. 0% needed mechanical ventilation.	
**Incidence venous thromboembolism**						
-	-	-	-	-	-	
**Incidence secondary infections**						
-	-	-	-	-	-	
**Incidence renal impairment**						
-	-	-	-	-	-	
**Long-term lung function data**						
-	-	-	-	-	-	

a) Prospective single-center cohort study with 114 immunocompromised patients including 16 LTR. Sotrovimab was initially used for patients requiring hospital admission and later in 14 outpatient LTR immediately upon COVID-19 diagnosis. [*] It is unclear how many of the 16 LTR, of whom 11 required hospitalization, were treated with sotrovimab upon hospitalization. COVID variant: 46% Omicron (mainly BA.1).

COVID-19: coronavirus 2019 disease, HFNC: high-flow nasale canula, MV: mechanical ventilation, NIV: non-invasive ventilation.

Solid organ transplant outcomes from studies that included lung transplant recipients

Bibliography:

a) Cochran W, Langlee J, Barker L, et al. Short-term outcomes in a nurse coordinator–led and nurse practitioner–led Sotrovimab initiative for solid organ transplant recipients during the omicron surge. Transplantation. 2022;106(9).
b) Hedvat J, Lange NW, Salerno DM, et al. COVID-19 therapeutics and outcomes among solid organ transplant recipients during the Omicron BA.1 era. Am J Transplant. 2022;22:2682-88.
c) Rasmussen LD, Lebech A-M, Øvrehus A, et al. Experience with sotrovimab treatment of SARS-CoV-2-infected patients in Denmark. Br J Clin Pharmacol. 2023;89(6):1820-33.
d) Solera JT, Árbol BG, Alshahrani A, et al. Impact of vaccination and early monoclonal antibody therapy on coronavirus disease 2019 outcomes in organ transplant recipients during the Omicron Wave. Clinical Infectious Diseases. 2022;75(12):2193-200.
e) Yetmar ZA, Beam E, O'Horo JC, et al. Outcomes of bebtelovimab and sotrovimab treatment of solid organ transplant recipients with mild-to-moderate Coronavirus disease 2019 during the omicron epoch. Transplant Infectious Disease. 2022;24(4).

Quality assessment			N° of patients		Effect	
N° of studies	Study design	Risk of bias	Intervention	No intervention	Relative	Absolute
**Overall mortality**						
5[a-e]	Observational	Intermediate	1314	262*		Two studies with control groups reported reduced mortality in the sotrovimab group (0/51 (0%) vs. 3/75 (4%) and 0/106 vs. 12/187 (6%)). The other studies also had a low mortality rate (0/88 (0%), 9/800 (1%) and 2/269 (0.7%)).<br>Hedvat et al. reported an association with lower risk of 30-day hospitalization and death and sotrovimab (aRR 0.15, CI95% 0.05–0.47). Rasmussen et al. reported that a delay in time of sotrovimab admission (≤ 3 vs > 3 days after testing positive) was associated with risk of death (multivariate HR: 4.88, CI95%: 1.27-18.73).
**COVID-19-related mortality**						
3[a,b,d]	Observational	Intermediate	245	75**		Lower mortality in the sotrovimab group (0/51, 0%) than the control group (3/75, 4%) in the study by Hedvat et al. Also in the other two studies, no patients treated with sotrovimab died due to COVID-19 (0/88 and 0/106).
**Hospital admission**						
5[a-e]	Observational	Intermediate	1314	262*		The studies with the control groups reported a lower incidence of hospitalization in the sotrovimab group (17/106 (16%) vs. 52/187 (28%), RR 0.58 (95%CI 0.35-0.94, p=0.33), and 6/51 (12%) vs. 25/75 (33%) for 30-day hospitalization or death from any cause (p=0.009) or 5/51 (10%) vs. 23/75 (31%) for 30-day hospitalization or death from COVID-19 (p=0.007)). In the latter study, sotrovimab was associated with a lower risk of 30-day hospitalization or death (aRR 0.15, CI95% 0.05–0.47).<br>Hospital admission ranged from 3% to 23% in the other studies (8/269, 9/88 and 183/800).<br>Solera et al. reported median stay of 4 days (IQR 3-7) vs. 7 days in control SOTR (IQR 5-16) (p=0.002).
**ICU admission**						

2d[,e]	Observational	Intermediate	375?	187[***]	No SOTR treated with sotrovimab (0/106) were admitted to ICU vs. 10% (18/187) in the control group in Solera et al. ICU admission was 1% (3/269) in Yetmar et al.
**Additional respiratory support (HFNC, NIV, MV)**					
3[a,b,d]	Observational	Intermediate	245	262[*]	In the studies with control groups, no SOTR treated with sotrovimab required mechanical ventilation vs. 5-8% in the controls (0/51 (0%) vs. 4/75 (5%) and 0/106 vs. 14/187 (8%)). In the other study, 0/88 (0%) required high-flow oxygen therapy, non-invasive ventilation or mechanical ventilation.<br>Solera et al. reported a RR 0.24 (95% CI, 0.1 to 0.59) of sotrovimab for supplemental oxygen treatment. NNT preventing supplemental oxygen treatment of 6.64 (95% CI, 4.56 to 13.66). Significant after adjusting age/ transplant type/number of vaccines and comorbidities (p=0.026).
**Incidence venous thromboembolism**					
1[d]	Observational	Intermediate	106	187[***]	1% (2/187) SOTR without sotrovimab suffered a pulmonary embolism.
**Incidence secondary infections**					
1[d]	Observational	Intermediate	106	187[***]	8/106 (8%) in sotrovimab group vs. 28/187 (15%) in control group (7% of SOTR with sotrovimab had a bacterial pneumonia, 1% a COVID-associated pulmonary aspergillosis and 0% a bloodstream infection vs. 9% (p=0.51), 2% (p=1) and 4%, respectively, in control SOTR).
**Incidence renal impairment**					
2[a,d]	Observational	Intermediate	157	262	10% of SOTR with sotrovimab suffered from acute kidney injury (AKI) in Hedvat et al. compared to 28% in SOTR without sotrovimab treatment (p=0.17). Solera et al. reported 13% of AKI in the sotrovimab group vs 21% in controls (p=0.12).
**Long-term lung function data**					
-	-	-	-	-	-

a) Retrospective single-center cohort study with 154 SOTR, of whom 24 LTR. 51 SOTR were treated with sotrovimab (including 4 LTR) upon COVID-19 diagnosis, 28 SOTR with nirmatrelvir/ritonavir and 75 SOTR received no SARS-CoV-2-specific treatment. COVID-19 variant: Omicron BA.1.

b) Retrospective single-center cohort-study with 88 SOTR including 18 LTR. COVID-19 variant: Omicron BA.1.

c) Prospective single-center cohort study with 300 SOTR, of whom 62 LTR. 106 SOTR were treated with sotrovimab (including 34 LTR). COVID-19 variant: Omicron BA.1.

d) Nationwide, population-based cohort study with 2933 high-risk patients including 800 SOTR (with 49 LTR and 2 heart-lung transplants). Sotrovimab was mainly given as outpatient treatment (88% of SOTR). COVID-19 variant: majority of all patients had Omicron BA.2 (54%), followed by Omicron BA.1 (13%) and Delta (10%).

e) Retrospective multicenter cohort study with 361 SOTR, including 21 LTR. 269 SOTR (17 LTR) were treated with sotrovimab. COVID-19 variant: Omicron B.1.1.527 and BA.2.

[*] Studies of Hedvat et al. (n=75) and Solera et al. (n=187)

[**] Study of Hedvat et al. including 75 SOTR without additional treatment

[***] Study of Solera et al. including cohort of 187 SOTR

COVID-19: coronavirus 2019 disease, HFNC: high-flow nasale canula, MV: mechanical ventilation, NIV: non-invasive ventilation.

Casirivimab-imdevimab

Solid organ transplant outcomes from studies that included lung transplant recipients

Bibliography:

a) Sarrell BA, Bloch K, El Chediak A, et al. Monoclonal antibody treatment for Covid-19 in solid organ transplant recipients. Transplant Infectious Disease. 2021;24(1).
b) Yetmar ZA, Beam E, O'Horo JC, et al. Monoclonal antibody therapy for COVID-19 in solid organ transplant recipients. Open Forum Infectious Diseases. 2021;8(6).

Quality assessment			N° of patients		Effect	
N° of studies	Study design	Risk of bias	Intervention	No intervention	Relative	Absolute
**Overall mortality**						
2[a-b]	Observational	Intermediate	40	72*	No patients who received casirivimab-imdevimab died (0/18 and 0/22), versus 2/72 (3%) in the control group in Sarrell et al.	
**COVID-19-related mortality**						
2[a-b]	Observational	Intermediate	40	72	No patients who received casirivimab-imdevimab died due to COVID-19 (0/18 and 0/22), versus 1/72 (1%) in the control group in Sarrell et al.	
**Hospital admission**						
2[a-b]	Observational	Intermediate	40	72*	In Sarrell et al., 0/22 treated with casirivimab-imdevimab were admitted for COVID-19-directed therapy vs. 11/72 (15%) in the control group (with a total admission rate of 14/72 or 19%). In the other study, 1/18 (6%) was admitted.	
**ICU admission**						
2[a-b]	Observational	Intermediate	40	72*	No patients treated with casirivimab-imdevimab were admitted to ICU vs. 1/72 (1%) in the control group in Sarrell et al.	
**Additional respiratory support (HFNC, NIV, MV)**						
2[a-b]	Observational	Intermediate	40	72*	a) 0/18 (0%).<br>b) 0/22 (0%) vs. 0/72 (0%) in controls required mechanical ventilation.	
**Incidence venous thromboembolism**						
-	-	-	-		-	
**Incidence secondary infections**						
-	-	-	-		-	
**Incidence renal impairment**						
1[a]	Observational	Intermediate	22	72	0/22 (0%) vs. 1/11 (9%) hospitalized (or 1/72 (1%) overall) controls needed renal replacement therapy.	
**Long-term lung function data**						
-	-	-	-		-	

a) Retrospective single-center cohort study with 165 patients, including 13 LTR and 72 controls, of whom 22 were treated with casirivimab-imdevimab. COVID-19 variant most likely alpha variant (B1.1.7).
b) Retrospective single-center cohort study with 73 SOTR, including 2 LTR, of whom 18 were treated with casirivimab-imdevimab. COVID-19 variant most likely alpha variant (B1.1.7).

[*] Study of Sarrell et al. with 72 SOTR who did not receive monoclonal antibody therapy.

COVID-19: coronavirus 2019 disease, HFNC: high-flow nasale canula, MV: mechanical ventilation, NIV: non-invasive ventilation.

Bamlanivimab

Solid organ transplant outcomes from studies that included lung transplant recipients

Bibliography:

a) Sarrell BA, Bloch K, El Chediak A, et al. Monoclonal antibody treatment for Covid-19 in solid organ transplant recipients. Transplant Infectious Disease. 2021;24(1).

b) Yetmar ZA, Beam E, O'Horo JC, et al. Monoclonal antibody therapy for COVID-19 in solid organ transplant recipients. Open Forum Infectious Diseases. 2021;8(6).

Quality assessment			N° of patients		Effect	
N° of studies	Study design	Risk of bias	Intervention	No intervention	Relative	Absolute
**Overall mortality**						
2[a,b]	Observational	Intermediate	126	72*	No patients who received bamlanivimab died (0/55 and 0/71), versus 2/72 (3%) in the control group in Sarell et al.	
**COVID-19-related mortality**						
1[a]	Observational	Intermediate	126	72	No patients who had bamlanivimab died due to COVID-19 (0/55 and 0/71), versus 1/72 (1%) in the control group in Sarell et al.	
**Hospital admission**						
2[a,b]	Observational	Intermediate	126	72*	Hospital admission due to COVID-19 ranged from 11% (8/71) - 13% (7/55) for SOTR treated with bamlanivimab. In the study of Sarell et al., 19% (14/72) of SOTR without monoclonal antibody therapy were hospitalized,11/72 (15%) for COVID-19-directed therapy (p=0.161).	
**ICU admission**						
2[a,b]	Observational	Intermediate	126	72*	In the study of Sarell et al., 2/71 (3%) were admitted to ICU compared to 1/72 (1%) patient in control cohort. In the other study, no patients (0/55) were admitted to ICU due to COVID-19.	
**Additional respiratory support (HFNC/NIV/MV)**						
2[a,b]	Observational	Intermediate	126	72*	In Yetmar et al. noted no high-flow oxygen therapy, non-invasive or mechanical ventilation. In Sarrell et al., 1/71 (1%) SOTR treated with bamlanivimab was mechanically ventilated vs. 0/72 (0%) in the controls.	
**Incidence venous thromboembolism**						
-	-	-	-	-	-	
**Incidence secondary infections**						
-	-	-	-	-	-	
**Incidence renal impairment**						
1[a]	Observational	Intermediate	71	72*	Of the hospitalized patients, 6/8 (75% or 8% overall) treated with bamlanivimab had acute kidney injury with 0% (0/71) requiring renal replacement therapy compared to 4/11 (36% or 6% overall) in the control group with 1% overall (1/72) requiring renal replacement therapy.	
**Long-term lung function data**						

-	-	-	-	-

a) Retrospective single-center cohort study with 165 patients, including 13 LTR and 72 controls, of whom 71 were treated with bamlanivimab. COVID-19 variant most likely alpha variant (B1.1.7).
b) Retrospective single-center cohort study with 73 SOTR, including 2 LTR, of whom 55 were treated with bamlanivimab. COVID-19 variant most likely alpha variant (B1.1.7).

[*] Study of Sarrell et al. with 72 SOTR who did not receive monoclonal antibody therapy.

COVID-19: coronavirus 2019 disease, HFNC: high-flow nasale canula, MV: mechanical ventilation, NIV: non-invasive ventilation.

Bebtelovimab

Solid organ transplant outcomes from studies that included lung transplant recipients

Bibliography:

a) Cochran W, Salto-Alejandre S, Barker L, et al. Covid-19 outcomes in solid organ transplant recipients who received Tixagevimab-cilgavimab prophylaxis and/or Bebtelovimab treatment in a nurse-driven monoclonal antibody program during the Omicron Surge. Transplantation. 2022;107(2).

b) Yetmar ZA, Beam E, O'Horo JC, et al. Outcomes of bebtelovimab and sotrovimab treatment of solid organ transplant recipients with mild-to-moderate Coronavirus disease 2019 during the omicron epoch. Transplant Infectious Disease. 2022;24(4).

Quality assessment			N° of patients		Effect	
**N° of studies**	**Study design**	**Risk of bias**	**Intervention**	**No intervention**	**Relative**	**Absolute**
**Overall mortality**						
2[a,b]	Observational	Intermediate	237	-	0.7% (1/145) – 2% (2/92).	
**COVID-19-related mortality**						
1[b]	Observational	Intermediate	92	-	At least 1 SOTR (1/2 who died, 1% of overall cohort) died due to COVID-19.	
**Hospital admission**						
2[a,b]	Observational	Intermediate	237	-	3% (3/92) – 12% (18/145).	
**ICU admission**						
1[b]	Observational	Intermediate	92	-	0% (0/92).	
**Additional respiratory support (HFNC, NIV, MV)**						
1[a]	Observational	Intermediate	145	-	0.7% (1/145) was mechanically ventilated.	
**Incidence venous thromboembolism**						
-	-	-	-		-	
**Incidence secondary infections**						
-	-	-	-		-	
**Incidence renal impairment**						
-	-	-	-		-	
**Long-term lung function data**						
-	-	-	-		-	

a) Retrospective single-center study of 350 SOTR, of whom 145 were treated with bebtelovimab (including 18 LTR). COVID-19 variant: Omicron BA.2 and BA.5.

b) Retrospective multicenter cohort study with 361 SOTR, 92 SOTR (including 4 LTR) were treated with Bebtelovimab. COVID-19 variant: Omicron B.1.1.527 and BA.2.

COVID-19: coronavirus 2019 disease, HFNC: high-flow nasale canula, MV: mechanical ventilation, NIV: non-invasive ventilation.
